# Prevalence of anisometropia and influencing factors among school-age children in Nantong, China: a cross-sectional study

**DOI:** 10.3389/fpubh.2023.1190285

**Published:** 2023-06-15

**Authors:** Yue Zhou, Xiao Fang Zhang, Xiao Juan Chen, Min Wang, Jian Ru Cai, Yao Jia Xiong, Yu Song, Zhi Min Sun

**Affiliations:** ^1^Department of Ophthalmology, Second Affiliated Hospital of Nantong University, Nantong, Jiangsu, China; ^2^Department of Nantong Fifth People’s Hospital, Nantong, Jiangsu, China

**Keywords:** Anisometropia, prevalence, school age children, risk factors, scoliosis

## Abstract

**Objective:**

To investigate the prevalence of anisometropia and associated parameters among school-aged children in Nantong, China.

**Methods:**

This school-based, cross-sectional study examined students from primary schools, junior high schools, and senior high schools in an urban area of Nantong, China. Univariate and multivariate logistic regression analyses were used to investigate the specific correlations between anisometropia and related parameters. Non-cycloplegic autorefraction was assessed for each student. Anisometropia was defined as the spherical equivalent refraction (SE) difference ≥ 1.0 D between eyes.

**Results:**

A total of 9,501 participants were validated for analyses, of which 53.2% (*n* = 5,054) were male, and 46.8% (*n* = 4,447) were female. The mean of age was 13.32 ± 3.49 years, ranging from 7–19 years. The overall prevalence of anisometropia was 25.6%. Factors such as myopia, scoliosis screening positive, hyperopia, female sex, older age, and higher weight had a significantly higher risk of anisometropia (*p* < 0.05).

**Conclusion:**

There was a high prevalence of anisometropia in school-age children. Some physical examination parameters are closely related to children’s anisometropia, especially myopia and scoliosis. Preventing myopia and controlling its progression may be the most important ways to reduce the prevalence of anisometropia. Correcting scoliosis may be an important factor in controlling the prevalence of anisometropia, and maintaining good reading and writing posture may be helpful in controlling the prevalence of anisometropia.

## 1. Introduction

Anisometropia involves asymmetry between eyes in the refractive state (1), it affects around 10% of the population in early adulthood (2). This phenomenon is manifested in eyes of individuals who may have similar sociodemographic, environmental, and genetic effects, and showing asymmetric eye growth (3). Anisometropia can be associated with strabismus, amblyopia, hyperopia, intolerance to glasses, and deepening of myopia (4). A previous study reported that the presence of anisometropia increased the risk of poor stereoscopic acuity by 6.73 times (5). It is one of the main causes of monocular vision loss, and unilateral amblyopia increases the risk of vision loss during a patient’s life (6, 7). The asymmetry of visual experience in childhood may change the growth of the ocular axis. Considering that the baseline spherical equivalent refraction (SE) of the two eyes may be different, and that differences between the two eyes may be caused by various reasons (8–11), the length of the eye axis may be asymmetrically prolonged, which may lead to anisometropia.

At present, research on children’s refractive errors and physical development status mainly focuses on the correlation between myopia and anthropometric measures (12–17). Although studies have described the refractive status, eye structure, demography, and lifestyle of patients with anisometropia (5, 18–23), there is still a lack of epidemiological research on the relationship between anisometropia of school age children and related parameters of children’s growth and development.

The specific cause of anisometropia is not clear. In China, scoliosis is the main type of spinal curvature abnormality (24, 25). In recent years, some regions in China have included scoliosis screening in students’ routine physical examinations. Considering that it has been confirmed that scoliosis is associated with poor reading and writing posture (26, 27), and that poor reading and writing posture may cause anisometropia (28), we also included scoliosis in the study of anisometropia.

Knowing the status of children’s’ anisometropia and identifying the causes will help in preventing permanent damage to binocular and stereoscopic vision. In the present study, we therefore analyzed the baseline data of routine physical examinations of students in 2022, to report the prevalence of refractive anisometropia and evaluate the association between other physical examination parameters and refractive anisometropia among school-age children.

## 2. Materials and methods

### 2.1. Design and subjects

This school-based study was designed to investigate the refractive status in schoolchildren in 2022 in Nantong, a moderately sized city on the east coast of China. According to data from the census of 2020, the total population of Nantong was 77,266,000. This study was approved by the ethics committee of the Second Affiliated Hospital of Nantong University, China (approval number: 2020KT068). All protocols used in this study followed the tenets of the Declaration of Helsinki. Written informed consent was obtained from parents of the participants before being enrolled.

Based on previous research, a stratified cluster sampling method was used (29). The cluster was stratified by grade and age to ensure that all age groups from 7 to 19 years of age were included in the study. Classes in each grade were selected by simple random sampling, and all students in these classes were required to participate in the study. The sampling framework was based on the statistics of classes of specific grades in schools at all levels. Previous studies have shown that the prevalence of anisometropia was stable at about 7% from 1 year of age to teenage years (5). To achieve a power of 80%, the sample size was calculated using the formula, *n* = t^2^pq/d^2^, assuming a design effect of 1.5 due to cluster sampling and a nonresponse rate of 5% [*t* = 2 for a 95% confidence interval (CI), *q* = 1−P, d = 0.1 P]. The total sample size was at least 8,391. To ensure better multifactor analyses (including some factors that previously were rarely studied), more samples were included in the protocol. This research included primary school, junior high school, and senior high school students in Nantong City as research subjects in the urban area of Nantong, from 27 schools (nine primary schools, nine middle schools, and nine high schools), which participated in the survey. At least two classes were randomly selected from each grade of each school to ensure that no less than 80 students were selected at a time. This sample size was sufficient to detect risk factors using multivariate analyses.

Before the study began, researchers visited and arranged each venue to standardize the lighting and test distance. To minimize interference and limit the number of students examined, closed classrooms were used to facilitate testing. Autorefractors were calibrated every day. Students with current corneal refractive therapy were asked to wear spectacle glasses on the day of testing. The proportion of children who volunteered to participate in the invitation was 96.3%.

### 2.2. Ophthalmic examinations

During the visual examination, well-trained investigators performed eye examinations, and non-cycloplegic refraction was measured using three repeated measurements using an autorefractor (WSRMK-8000; Biobase, Shandong, China). The average data of three repeated measurements were used for analysis. Refractive error was measured three times starting with the right eye; if any two of the three results were greater than 0.50 D (diopters), additional examinations were conducted at the same visit. Vision measurement started with the right eye, and uncorrected visual acuity was measured by a standard logarithmic liquid crystal tumbling E chart (WSVC-100; Qingda Optometry, Berkeley, CA, United States) at 5 m. The best-corrected visual acuity was corrected according to the autorefractor results. Refinement of the sphere, cylinder, and axis was done to achieve the best-corrected visual acuity. Spherical equivalent refraction (SE) was calculated using the cylindrical degree and spherical degree as follows: SE = cylindrical degree × 0.5 + spherical degree. Similar to previous epidemiological studies, the present study used the Refractive Error Study in Children surveys (30, 31). Anisometropia was defined as the spherical equivalent refraction (SE) difference ≥ 1.0 D between eyes. To minimize the potential impact of spurious associations between anisometropia and ametropia, subjects were categorized according to the SE in less ametropic eyes. Myopia was defined as a spherical equivalent of ≤−0.5 D. Hyperopia was defined as an SE > 0.5 D. Emmetropia was defined as −0.5 D < SE ≤ 0.5 D. Low myopia was defined as −3.0 D < SE ≤ −0.5 D. Moderate myopia was defined as −6.0 D < SE ≤ −3.0 D. High myopia was defined as a SE < −6.0 D. The degree of anisometropia was categorized into mild (SE difference ≥ 1.0 D and < 2.0 D), moderate (SE difference ≥ 2.0 D and < 3.0 D), and severe (SE difference ≥ 3.0 D). Those students who were suspected of ocular abnormality were referred to subspecialists for further investigation.

### 2.3. Scoliosis examination

An experienced screening team, comprising of 10 spine surgeons, rehabilitation physicians, therapists, and nurses, from the Second Affiliated Hospital of Nantong University performed the school-based screening. Boys and girls received relevant examinations. Inspectors could only assume their posts after receiving unified training and passing the necessary examinations. Participants with spine and chest deformities (including treatment with a brace), musculoskeletal anomalies, neurological disorders, and operation histories were questioned and excluded from the study. According to the national standard “Screening of Children and Adolescents with Abnormal Spinal Curvature” (GB/T 16133–2014), they had passed the general examination, forward flexion test, spine movement test, and prone test, and the scoliosis measuring instrument was used to screen scoliosis in schools. The results were divided into no side bending, side bending degree 1, side bending degree 2, and side bending degree 3. Students with positive scoliosis screening were registered in this study and recommended to go to a specialized hospital for further examinations.

Other conventional physical examinations were conducted by physicians from tertiary hospitals, including height, weight, and blood pressure. In addition, basic information such as sex and age were recorded. The height was determined to the nearest 0.1 cm in a standardized manner without shoes. Weight was measured to the nearest 0.1 kg without thick clothes.

### 2.4. Statistical analysis

Data from school-age children enrolled in 2022 who had completed the study were analyzed. Data were analyzed using SPSS statistical software for Windows, version 22 (SPSS, Chicago, IL, United States). Correlations between anisometropia and various parameters considered in this study were then determined. The differences between groups in terms of refractive status and physical examination parameters were compared using chi-square or independent *t*-tests, as appropriate. The chi-squared test was used for disordered enumeration data, the rank-sum test was used for orderly enumeration data, and the independent samples *t-*tests were used for measurement data. The polynomial linear correlation in one-way ANOVA was used for the trend test (Ptrend). Univariate and multivariate logistic regression analyses were used to investigate the specific correlation between anisometropia and related parameters. The odds ratio (OR) and 95% confidence interval (CI) for the associated factors were then calculated. Factors with an OR < 1 were regarded to be protective against anisometropia, whereas those with an OR > 1 were considered to be risk factors for anisometropia. Continuous variables are expressed as the mean ± standard deviation, and categorical variables are expressed as percentages. A value of *p* < 0.05 was considered statistically significant.

## 3. Results

A total of 9,864 students were invited to participate in the study. The completion percentage of students out of all schools was 6.5%. A total of 9,501 students were finally recruited into the statistical analysis. The distributions of basic demographic and ocular parameters are shown in Table 1. Of 9,501 students, 53.2% (n = 5,054) were male, and 46.8% (n = 4,447) were female. The mean of age was 13.31 ± 3.47 years, ranging from 7–19 years. The prevalence of anisometropia was not related to sex (x^2^: 3.14, *p* = 0.077). The prevalence of anisometropia in students with scoliosis screening positive (45.6%) was significantly higher than that in students without scoliosis screening positive (24.7%) (x^2^: 83.64, *p* < 0.001). In addition, Table 1 shows that anisometropia was related to refractive state, age, height, weight, systolic blood pressure, and diastolic blood pressure (all, *p* < 0.001).

**Table 1:** Characteristics of participants.

Characteristic	Total (*n* = 9,501)	With anisometropia (*n* = 2,428)	Without anisometropia (*n* = 7,073)	*p* value
Sex, n (%)				0.077
Male	5,054 (53.2)	1,254(51.6)	3,800(53.7)	
Female	4,447 (46.8)	1,174(48.4)	3,273(46.3)	
Refractive state, *n* (%)				0.000
Emmetropia	1,650 (17.4)	76(3.1)	1,574(22.3)	
Hyperopia	271 (2.9)	48(2.0)	223(3.2)	
Myopia	7,580 (79.8)	2,304(94.9)	5,276(74.6)	
Scoliosis screening positive, *n* (%)				0.000
Yes	382(4.0)	174(7.2)	208(2.9)	
No	9,119(96.0)	2,254(92.8)	6,865(97.1)	
Age (years)	13.31 ± 3.47	14.65 ± 3.06	12.85 ± 3.48	0.000
Height (cm)	151.00 ± 17.59	157.66 ± 14.77	148.72 ± 17.90	0.000
Weight (kg)	47.37 ± 18.47	54.02 ± 18.11	45.09 ± 18.05	0.000
Systolic blood pressure (mmHg)	112.31 ± 12.55	115.18 ± 12.66	111.33 ± 12.37	0.000
Diastolic blood pressure (mmHg)	63.16 ± 8.20	64.50 ± 7.94	62.70 ± 8.23	0.000

**Figure 1:**
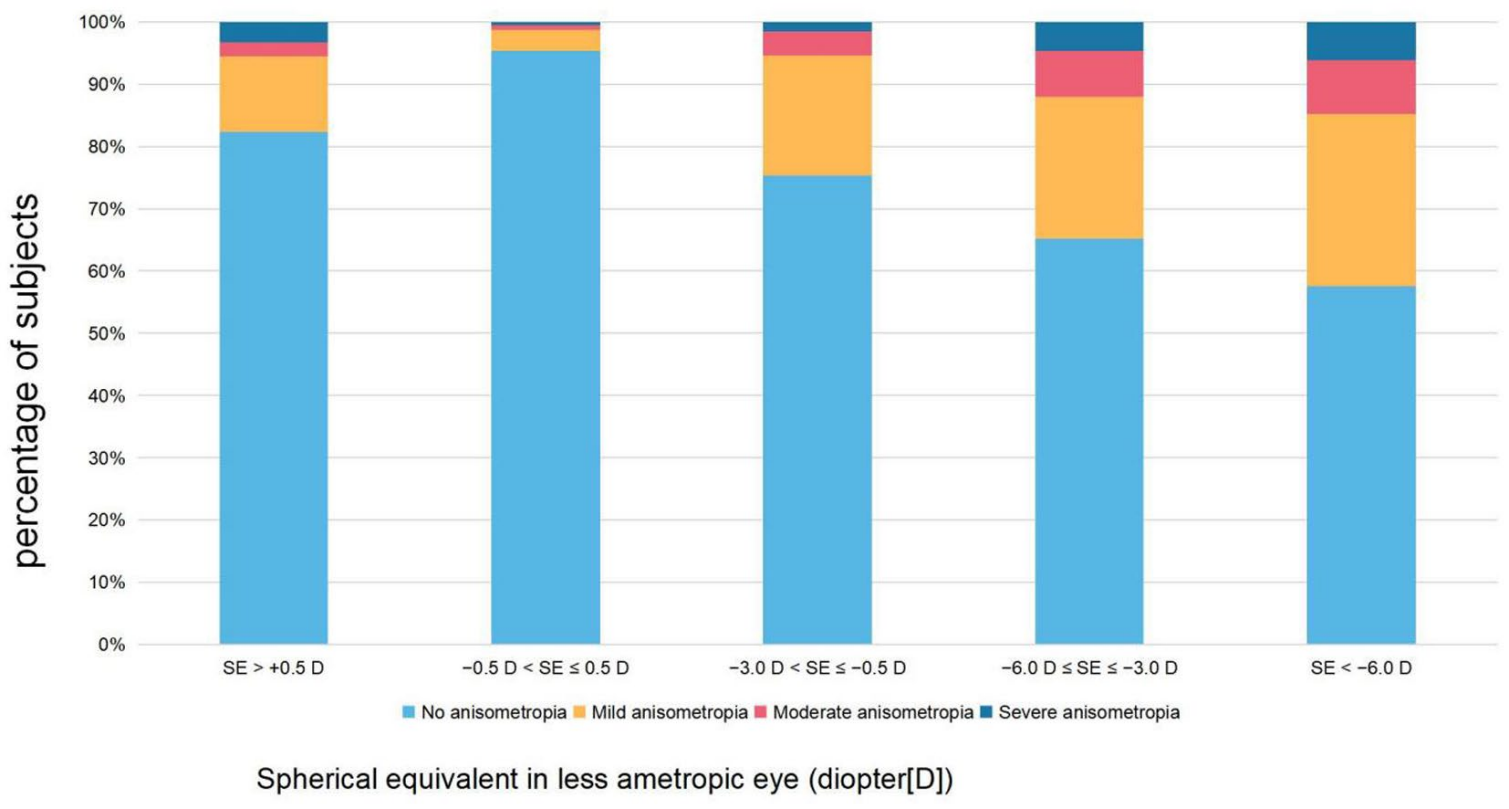
The proportion of different severity levels of refractive anisometropia by refractive groups.

As illustrated in Table 1 and Figure 1, anisometropia was more prevalent in the myopic and hyperopic groups (30.4 and 17.7%, respectively) than in the emmetropic group (4.6%). In the population with myopia, as the degree of myopia deepened, the proportion of anisometropia also increased. The prevalence of anisometropia in the low myopia group was 24.7%, in the moderate myopia group it was 34.9%, and even reached 42.4% in the high myopia group. The proportion of anisometropia ≥2.0 D in the emmetropia group was 1.3%, in the low myopia group it was 5.4%, in the moderate myopia group it was 12.1%, and reached 14.8% in the high myopia group.

**Figure 2:**
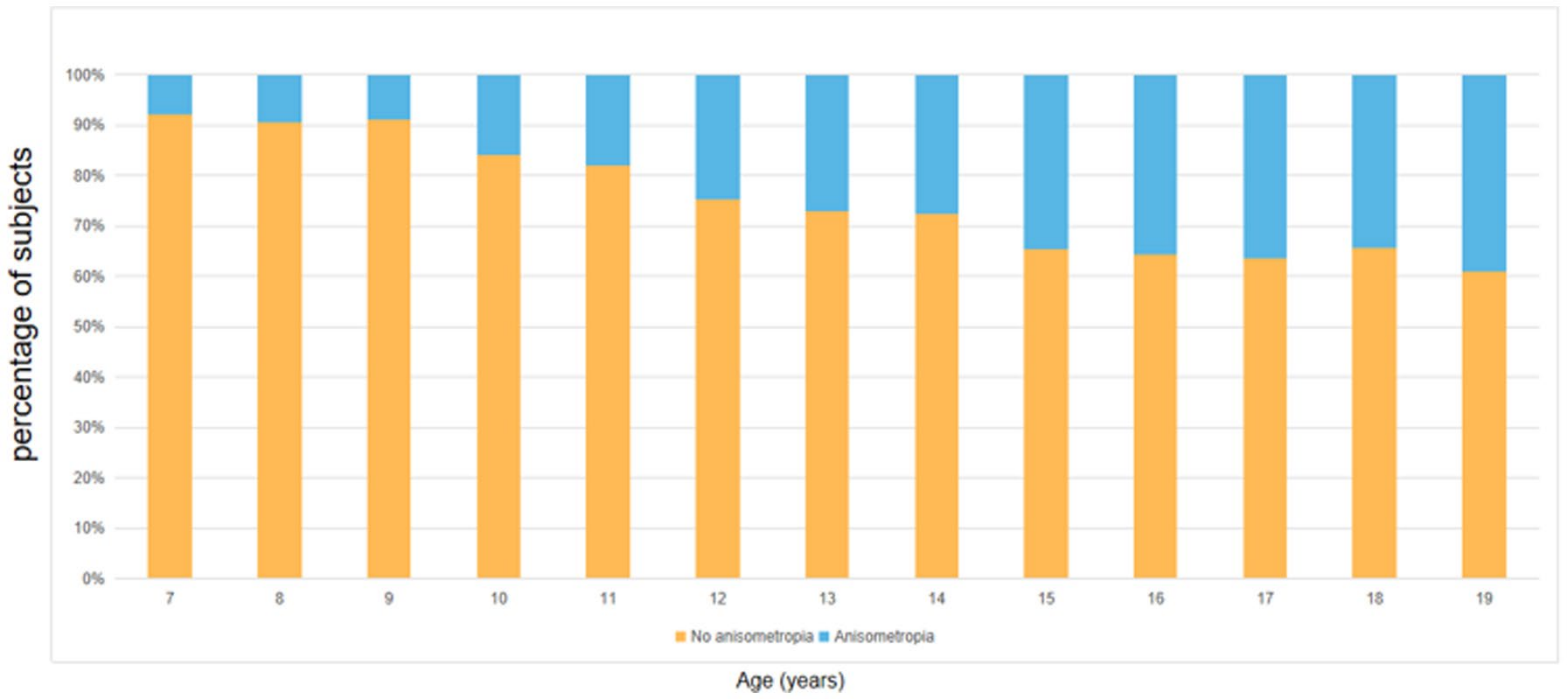
The proportion of different severity levels of anisometropia by age.

As illustrated in Table 2 and Figure 2, the overall prevalence of anisometropia was 25.6% (n = 2,428), and the mean SE difference between eyes was 0.72 ± 0.83 D. The overall prevalence of myopia was 79.8%. The prevalence of anisometropia gradually increased with age (Ptrend < 0.001). The prevalence of anisometropia at the age of 7 years was 7.8%, reaching 39.0% at the age of 19 years. The prevalence of myopia tended to increase with age (Ptrend < 0.001). The prevalence of myopia at the age of 7 years was 10.1%, reaching 89.7% at the age of 19 years. With increasing age, the difference in inter-eye SE gradually widened (Ptrend < 0.001).In addition, the prevalence of hyperopia tended to decrease with age (Ptrend < 0.001), and the prevalence of scoliosis screening positive did not change with age (Ptrend = 0.911).

**Table 2:** Prevalence of anisometropia, myopia and scoliosis screening positively stratified by age.

Age	*n*	Prevalence of anisometropia	Prevalence of myopia	Prevalence of scoliosis screening positive	Difference of inter-eye SE (D)	SE of the less ametropic eye (D)
7	217 (2.3%)	7.8%	10.1%	6.9%	0.38 ± 0.54	−0.11 ± 1.01
8	729 (7.7%)	9.3%	16.2	4.0%	0.41 ± 0.47	−0.43 ± 1.08
9	733 (7.7%)	9.0%	23.7%	4.1%	0.43 ± 0.50	−0.73 ± 1.28
10	824 (8.7%)	16.0%	36.8%	4.6%	0.50 ± 0.59	−1.08 ± 1.38
11	781 (8.2%)	18.1%	48.5%	4.6%	0.57 ± 0.64	−1.65 ± 1.75
12	810 (8.5%)	24.8%	63.8%	2.2%	0.71 ± 0.74	−2.21 ± 1.84
13	776 (8.2%)	27.1%	69.7%	3.7%	0.78 ± 0.93	−2.62 ± 1.97
14	828 (8.7%)	27.5%	74.4%	3.9%	0.80 ± 0.91	−2.97 ± 2.08
15	815 (8.6%)	34.7%	82.6%	3.2%	0.92 ± 1.03	−3.36 ± 2.09
16	800 (8.4%)	35.6%	82.6%	3.8%	0.88 ± 0.85	−3.59 ± 2.20
17	783 (8.2%)	36.4%	86.0%	3.8%	0.95 ± 1.02	−4.00 ± 2.43
18	776 (8.2%)	34.4%	88.1%	5.0%	0.87 ± 0.84	−4.25 ± 2.42
19	629 (6.6%)	39.0%	89.7%	4.8%	0.95 ± 0.96	−4.41 ± 2.52
Total	9,501 (100.0%)	25.6%	79.8%	4.0%	0.72 ± 0.83	−2.54 ± 2.37
χ 2 (F)		404.50	2320.44	0.01	504.12	4441.84
*p*-value		0.000	0.000	0.911	0.000	0.000

Results of the chi-square test for trend test (Ptrend).

Table 3 lists the results of univariate and multiple logistic regression analyses. After adjustment for other characteristics, the results suggested that females were more likely to suffer from anisometropia than males (OR: 1.25, 95% CI: 1.13–1.39, *p* < 0.001). Compared with students with emmetropia, students with hyperopia were 2.25 times more likely to suffer from anisometropia (OR: 2.25, 95% CI: 1.45–3.47, *p* < 0.001) and students with myopia were 6.40 times more likely to suffer from anisometropia (OR: 6.40, 95% CI: 5.01–8.18, *p* < 0.001). Compared with students with screening negative, students with scoliosis screening positive were 4.08 times more likely to suffer from anisometropia (OR: 4.08, 95% CI: 3.14–5.30, *p* < 0.001). In addition, anisometropia was independently associated with students with older age (OR: 1.05, 95% CI: 1.02–1.08, *p* < 0.05) and higher weight (OR: 1.01, 95% CI: 1.01–1.02, *p* < 0.001).

**Table 3:** Univariate and multivariate logistic regression analysis of anisometropia among all participants (*n* = 9,501).

Characteristic	Univariate analysis			Multivariate analysis		
	Crude OR	95% CI	*p* value	Adjusted OR	95% CI	*p* value
Sex						
Male	Reference			Reference		
Female	1.09	0.99–1.19	0.08	1.25	1.13–1.39	0.000
Refractive state						
Emmetropia	Reference			Reference		
Hyperopia	4.46	3.03–6.57	0.000	2.25	1.45–3.47	0.000
Myopia	9.04	7.15–11.44	0.000	6.40	5.01–8.18	0.000
Scoliosis screening positive						
Yes	2.55	2.07–3.13	0.000	4.08	3.14–5.30	0.000
No	Reference			Reference		
Age	1.17	1.15–1.19	0.000	1.05	1.02–1.08	0.002
Height	1.03	1.03–1.04	0.000	1.00	1.00–1.01	0.26
Weight	1.03	1.02–1.03	0.000	1.01	1.01–1.02	0.000
Systolic blood pressure	1.03	1.02–1.03	0.000	1.00	0.99–1.01	0.956
Diastolic blood pressure	1.03	1.02–1.03	0.000	1.00	0.99–1.01	0.749

OR = odds ratio; 95% CI = 95% confidence interval.

## 4. Discussion

The current study evaluated the prevalence and associated factors of anisometropia related to growth and development. This was a unique study involving anisometropia and growth and development of school-age children between 7 and 19 years of age. We found that anisometropia was related to myopia, scoliosis screening positive, hyperopia, female sex, older age, and higher weight.

Considering that most studies (32–34) focused on ≥1.00 D as the standard of anisometropia, we also used this critical point to assess its correlation with other parameters. Table 4 summarizes the main results of epidemiological studies on the prevalence of anisometropia in school aged children in recent years. There were differences in the prevalence of anisometropia in studies of different countries, races, and ages. In an epidemiological study of 2,090, 6–72 months-old children in Australia (23), the prevalence of anisometropia was 2.7%. In another epidemiological study of 1,765, 6-year-old children in Australia, the prevalence of anisometropia was only 1.6% (20). In Northern Ireland (35), the prevalence of anisometropia in an epidemiological study of 389 Caucasian children aged 6–7 years was 8.5%, and in 661 Caucasian children 12–13 years of age it was 9.4%. In Portugal (33), the prevalence of anisometropia varied from 2.9% in pre-school children to 9.4% in their 3rd study cycle. Deng & Gwiazda (36) found that when using a cutoff of 1.00 D SER for anisometropia, the prevalence were 2.0, 1.3, and 5.8% at 6 months, 5 years, and 12–15 years, respectively. In a large-scale school study in Taipei (19), 5.3% of 23,114 8-year-old children had anisometropia. In 2016, a school-based study conducted in Shandong, China (5) found that 7.0% of 6,025 school children aged 4–17 years had refractive anisometropia. In 2022, in another epidemiological survey of students 4–17 years of age in Shandong, China (37), the prevalence rose to 13.2%. In the current study, we found that the prevalence of anisometropia was relatively low in early school age children (7–9 years of age) (7.8–9.0%), but it increased to 16.0% at 10 years of age, and even to 39.0% at 19 years of age. In addition, the prevalence of anisometropia from 15 years of age was even higher than that of previous relevant studies on adults (1, 38). Compared with previous studies, such a high prevalence of anisometropia is rare. In fact, compared to previous studies (5, 35, 37), there is not much difference in the prevalence of anisometropia between 7 to 9 years of age. The sharp increase in the prevalence of anisometropia is worth noting.

**Table 4:** Epidemiological studies on the prevalence of refractive anisometropia.

Study (year)	Number of participant and area	Study design	Ethnicity	Age	Prevalence of anisometropia
Afsari et al. (23)	*n* = 2,090; Australia	Population-based	European-Caucasian (46.9%) East-Asian (20.2%), South-Asian (13.2%), Middle-Eastern (8.7%), Others/Mix (10.9%)	6–72 months	2.7%
Huynh et al. (20)	*n* = 1,765; Australia	Population-based	European white (63.6%) East Asian (17.1%)	6 years	1.6%
O’Donoghue et al. (35)	*n* = 1,053; Northern Ireland	Population-based	European-Caucasian	6–7 years 12–13 years	8.5% at 6–7 years, 9.4% at 12–13 years
Deng & Gwiazda (36)	*n* = 1,120; United States	Longitudinal	White	6 months 5 years 12 to 15 years	1.96% at 6 months, 1.27% at 5 years 5.7% at 12 to 15 years
Lee et al. (18)	*n* = 23,114; Taipei, China	Population-based	East Asian	8 years	5.3%
Hu et al. (5)	*n* = 6,025; Taipei, China	Population-based	East Asian	4 to 18 years	7.0% ± 0.3%
Nunes et al. (33)	*n* = 749; Portugal	Population-based	White	3 to 16 years	6.1%
Wu et al. (29)	*n* = 6,026; Shandong	Population-based	East Asian	4 to 17 years	7.0% ± 0.3%
Xu et al. (37)	*n* = 4,198; Shandong	Population-based	East Asian	4 to 17 years	13.2%

An interesting finding of this study was that scoliosis screening positive was one of the important factors of anisometropia. This has not been reported in previous studies. Scoliosis refers to deformity of the spine, with one or more segments of the spine bending to the side or accompanied by vertebral rotation. Recent studies (39, 40) reported that there may be millions of children with idiopathic scoliosis in China. To detect, diagnose, and treat early, and improve long-term prognoses, it is necessary to conduct relevant screening. Scoliosis can be divided into idiopathic scoliosis and non-idiopathic scoliosis, of which idiopathic scoliosis accounts for about 80% of patients. The prevalence of idiopathic scoliosis ranges from 2–16% (41–44). Diagnosis of scoliosis requires radiographic examination, and students with a Cobb angle of at least 10° are diagnosed as positive (45–47). In the current study, children with scoliosis screening positive were referred for further appointments, so we could not obtain additional relevant data. Although X-ray examinations were not used for final diagnoses in this study, the positive predictive value of idiopathic scoliosis has reached 78.4% in previous similar three-stage design examinations (24, 41). Furthermore, the main objective of this study was not to diagnose scoliosis, but to study the prevalence of anisometropia and its related parameters. It was therefore appropriate to use the parameters of suspected scoliosis for relevant studies. In the present study, the overall prevalence of positive screening for scoliosis in children and adolescents was 4.0%, similar to the study conducted in Zhejiang Province, China in 2019 (24). The results of this study showed that the prevalence of anisometropia in children with scoliosis screening positive was 4.08 times higher than that in children without scoliosis (OR: 4.08, 95% CI: 3.14–5.30, *p* < 0.001). Spinal scoliosis inevitably leads to poor reading and writing posture (26, 27). Pärssinen et al. (48) found that in school-aged children, the trend of myopia progression was closely related to steeper reading angle. When poor reading and writing posture persists, the refractive stimulation to both eyes vary. Assuming adaptation to fixed points is maintained, much of the peripheral field must be greatly out-of-focus. Hence, myopia and anisometropia would result (28). In previous studies on near work habits (18, 49, 50), including the age at which close working began, the distance between eyes and objects, the use of computers or mobile devices, and the average daily number near working activities, no correlation was found between near working and anisometropia. But the impact of poor reading and writing posture on refractive error has not been fully studied. Therefore, correcting scoliosis may be an important factor in controlling the prevalence of anisometropia, and maintaining good reading and writing posture may be helpful in controlling the prevalence of anisometropia.

However, the prevalence of scoliosis screening positive was only 4%, and it did not increase with age (Ptrend = 0.911), which could not explain the high prevalence of anisometropia and the characteristics of anisometropia with age. Similarly, although students with hyperopia were 2.25 times more likely to suffer from anisometropia (OR: 2.25, 95% CI: 1.45–3.47, *p* < 0.001), considering the trend of decreasing prevalence of hyperopia with age (Ptrend<0.001), it could not explain the high prevalence of anisometropia and the characteristics of anisometropia with age. According to current research, myopia is the most important factor associated with anisometropia. Students with myopia were 6.40 times more likely to suffer from anisometropia (OR: 6.40, 95% CI: 5.01–8.18, *p* < 0.001). And as shown in Table 2, both the prevalence of anisometropia and myopia showed a significant increasing trend with age (Ptrend<0.001).

In experimental anisometropia (34, 51, 52), anisometropia is highly correlated with the difference in axial length, mainly the growth of the posterior segment of the eye, which means that the induced anisometropia is essentially axial, and abnormal visual input of one eye may cause uneven axial elongation of both eyes. Similarly, significant correlation between anisometropia and axial length difference between eyes has also been found in human studies, and with an increase in myopia, both eyes gradually lose a balance of refractive errors (37, 38, 53, 54). In Sweden (55), the prevalence of myopia in 10-year-old children was 7.8; the prevalence of anisometropia was only 1%. In Dutch school children 11–13 years of age (56), the prevalence of myopia was 28% and the prevalence of anisometropia was 4.60%. In children aged 4 to 17 in Shandong, China (37), although there was no detailed list of the prevalence of myopia, the SE of the worse eye gradually decreased from 1.31 ± 0.77 to −3.92 ± 2.37 (Ptrend < 0.001), indicating the deepening of myopia. At this time, the prevalence of anisometropia increased from 1.1 to 28.4% (Ptrend < 0.001). In the present study, with increased age, the refractive status of many school-aged children shifted to myopia, and the prevalence of myopia increased from 10.1% at the age of 7 to 89.7% at the age of 19 (Ptrend < 0.001), which may have led to expansion of the range of refractive errors and significant differences between eyes. The prevalence of anisometropia in the low myopia group was 24.71%, in the moderate myopia group it was 34.87%, and even reached 42.41% in the high myopia group, which also indicated that the higher the degree of myopia, the greater the possibility of anisometropia. Therefore, preventing myopia and controlling its progression may be the most important ways to reduce the prevalence of anisometropia.

The impact of sex on anisometropia is controversial (57–60). A study conducted among high school students in Singapore (57) showed a higher prevalence of anisometropia among female participants. A study of participants aged ≥30 years in Bangladesh also showed similar results (58). In the present study, it was also found that females were more likely to suffer from anisometropia than males (OR: 1.25, 95% CI: 1.13–1.39, *p* < 0.001). In addition, some studies have reported the relationships between dominant eyes and anisometropic myopia, but there is still controversy (61, 62). Because no information about ocular advantages was collected in our study, we could not confirm a similar association.

### 4.1. Limitations

There were some potential limitations in the present study. First, the diopters used in this study were measured without cycloplegia, which ensured a high participation percentage. The prevalence of myopia may be overestimated in the absence of ciliary muscle paralysis (63). Second, we did not measure axial lengths to differentiate mechanisms related to anisometropia development during the growth of children’s’ eyes. Third, in this study, there was no final imaging diagnosis during the measurement of scoliosis. In addition, the use of autorefractometer may also lead to an overestimate of the prevalence of anisometropia. In the one hand, the autorefractometer measures the refraction close to the eye, which activates accommodation and tends to register measurements that overestimate myopia (64). On the other hand, when measuring each eye separately, that effect can be different between eyes, which overestimates anisometropia.

## 5. Conclusion

There was a high prevalence of anisometropia in school-age children in Nantong, China. The present study showed that parameters such as myopia, scoliosis, hyperopia, female sex, older age, and higher weight were significantly associated with a higher risk of anisometropia, especially myopia and scoliosis. Preventing myopia and controlling its progression may be the most important ways to reduce the prevalence of anisometropia. Correcting scoliosis may be an important factor in controlling the prevalence of anisometropia, and maintaining good reading and writing posture may helpful in controlling the prevalence of anisometropia.

## Data availability statement

The original contributions presented in the study are included in the article/supplementary material, further inquiries can be directed to the corresponding authors.

## Ethics statement

The studies involving human participants were reviewed and approved by the ethics committee of the Second Affiliated Hospital of Nantong University, China. The patients/participants provided their written informed consent to participate in this study.

## Author contributions

YZ, XZ, YS, and ZS were involved in the design of the study. XC, MW, JC, and YX were involved in data collection and data analysis. All authors contributed to the conception of the work by writing sections of the manuscript and drafting and revising it critically as well as final approval of the published version.

## Funding

This work was supported by Nantong Science and Technology Program (project number: MS2020035).

## Conflict of interest

The authors declare that the research was conducted in the absence of any commercial or financial relationships that could be construed as a potential conflict of interest.

## Publisher’s note

All claims expressed in this article are solely those of the authors and do not necessarily represent those of their affiliated organizations, or those of the publisher, the editors and the reviewers. Any product that may be evaluated in this article, or claim that may be made by its manufacturer, is not guaranteed or endorsed by the publisher.
